# Supplementary material for: Integrated analysis of the aqueous humor microbiome and lens capsule transcriptome in high myopia cataract: a pilot study
Source: Front Med (Lausanne). 2026 Jun 16;13:1845205. doi: 10.3389/fmed.2026.1845205 (PMC13314463; doi:10.3389/fmed.2026.1845205)
Supplement: Supplementary file 3 [file Data_Sheet_1.zip › 6.PICRUSt2/Meta_Statistic/C372089/Wilcoxon/KO_abundance.Wilcoxon.diff.top10.boxplot.pdf]

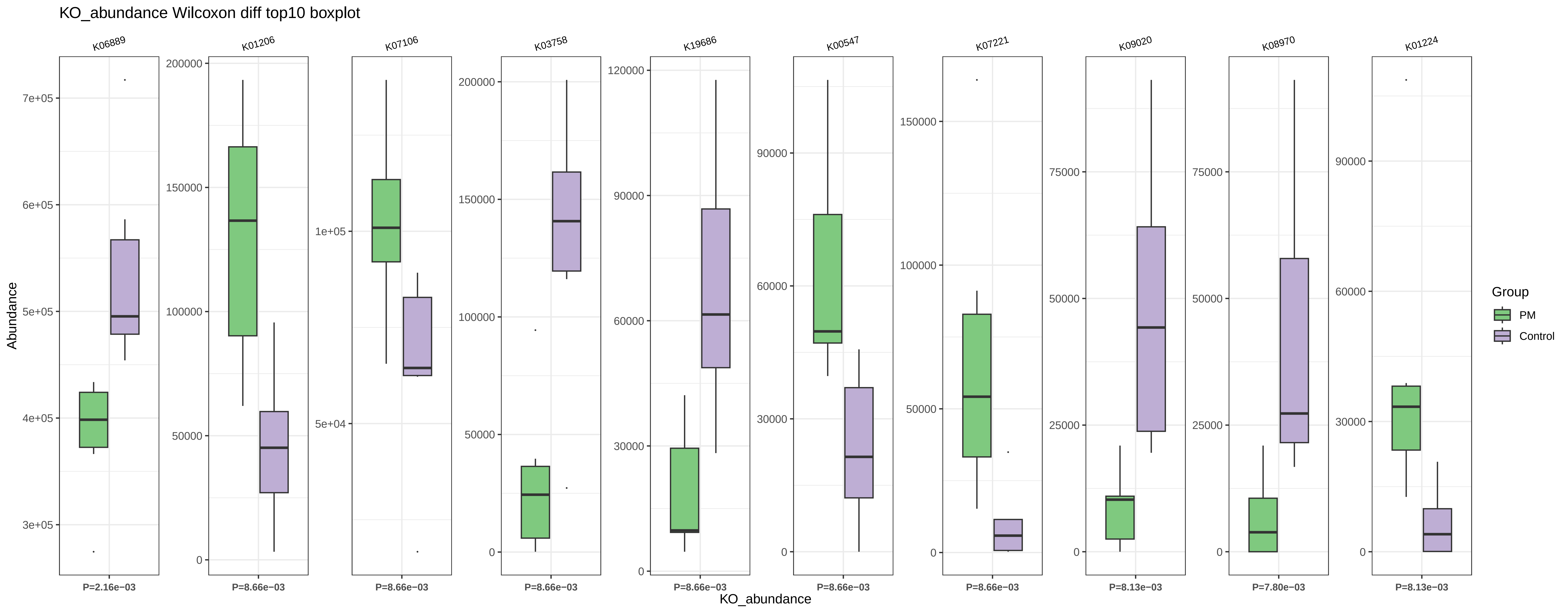
KO_abundance Wilcoxon diff top10 boxplot
K06889
K01206
K07106
K03758
K19686
K00547
K07221
K09020
K08970
K01224
Abundance
P=2.16e−03
P=8.66e−03
P=8.66e−03
P=8.66e−03
P=8.66e−03
P=8.66e−03
P=8.66e−03
P=8.13e−03
P=7.80e−03
P=8.13e−03
KO_abundance
Group
PM
Control
